# Lack of Causal Roles of Cannabinoid and Dopamine Neurotransmitter Systems in Orbitofrontal and Piriform Cortex in Fentanyl Relapse in Rats

**DOI:** 10.1523/ENEURO.0496-21.2022

**Published:** 2022-07-15

**Authors:** Sarah M. Claypool, Sana Behdin, Sarah V. Applebey, Javier Orihuel, Zilu Ma, David J. Reiner

**Affiliations:** Intramural Research Program, National Institute on Drug Abuse, National Institutes of Health, Baltimore, MD 21224

**Keywords:** addiction, cannabinoids, dopamine, opioid, relapse, self-administration

## Abstract

The orbitofrontal cortex (OFC) and piriform cortex (Pir) play a role in fentanyl relapse after food choice-induced voluntary abstinence, a procedure mimicking abstinence because of availability of alternative nondrug rewards. We used *in situ* hybridization and pharmacology to determine the role of OFC and Pir cannabinoid and dopamine receptors in fentanyl relapse. We trained male and female rats to self-administer food pellets for 6 d (6 h/d) and intravenous fentanyl (2.5 μg/kg/infusion) for 12 d (6 h/d). We assessed fentanyl relapse after 12 discrete choice sessions between fentanyl and food (20 trials/d), in which rats voluntarily reduced fentanyl self-administration. We used RNAscope to determine whether fentanyl relapse is associated with activity (indicated by *Fos*) in OFC and Pir cells expressing *Cnr1* [which encodes cannabinoid 1 (CB1) receptors] or *Drd1* and *Drd2* (which encode dopamine D1 and D2 receptors). We injected a CB1 receptor antagonist or agonist (0.3 or 1.0 μg AM251 or WIN55,212-2/hemisphere) into OFC or a dopamine D1 receptor antagonist (1.0 or 3.0 μg SCH39166/hemisphere) into Pir to determine the effect on fentanyl relapse. Fentanyl relapse was associated with OFC cells co-expressing *Fos* and *Cnr1* and Pir cells co-expressing *Fos* and *Drd1*. However, injections of the CB1 receptor antagonist AM251 or agonist WIN55,212-2 into OFC or the dopamine D1 receptor antagonist SCH39166 into Pir had no effect on fentanyl relapse. Fentanyl relapse is associated with activation of *Cnr1*-expressing OFC cells and *Drd1*-expressing Pir cells, but pharmacological manipulations do not support causal roles of OFC CB1 receptors or Pir dopamine D1 receptors in fentanyl relapse.

## Significance Statement

A previous study showed a role of orbitofrontal cortex (OFC) and piriform cortex (Pir) in fentanyl relapse after food choice-induced voluntary abstinence. Here, we aimed to determine the role of two neurotransmitter receptors, cannabinoid-1 receptors and dopamine D1 receptors in OFC and Pir, in fentanyl relapse. We found that fentanyl relapse is associated with activation of cells expressing these receptors in OFC and Pir, but causal pharmacological experiments do not support a role of OFC cannabinoid 1 receptors or Pir dopamine D1 receptors in fentanyl relapse.

## Introduction

A main feature of drug addiction is high rates of relapse during abstinence (Hunt et al., 1971; Sinha, 2011). A limitation of procedures modeling relapse in laboratory animals using extinction-reinstatement (Shalev et al., 2002; Kalivas and McFarland, 2003) or homecage forced abstinence (Venniro et al., 2016) is that the abstinence period is experimenter-imposed. In humans, abstinence is often voluntary because of either adverse consequences of drug use or availability of competing nondrug reinforcers (Epstein and Preston, 2003; Katz and Higgins, 2003).

**Figure 1.**
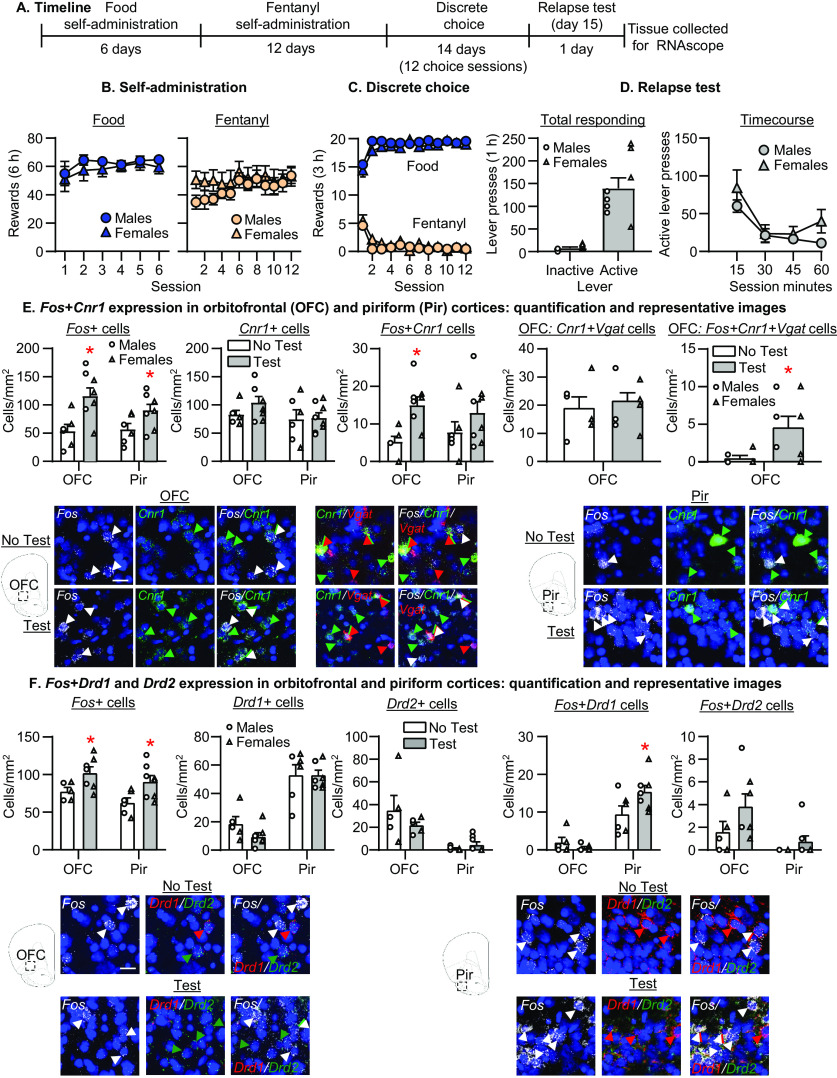
Effect of fentanyl relapse on activity in OFC and Pir cells expressing *Cnr1*, *Drd1*, and *Drd2*. ***A***, Timeline of experiment 1. ***B***, Self-administration. Number of reinforced responses (food: 5 pellets/reinforcer; fentanyl 2.5 μg/kg/infusion) during the 6-h sessions. ***C***, Discrete choice (voluntary reduction in self-administration). Number of food-reinforced responses and fentanyl infusions earned during the 3-h choice sessions (20 trials/session). ***D***, Relapse tests. Number of active and inactive lever presses during the 60-min test session (left) and the 15-min time course (right). ***E***, From left to right, Number of *Fos*+ cells per mm^2^, number of *Cnr1*+ cells per mm^2^, number of *Fos*+*Cnr1* double-labeled cells in OFC and Pir, number of *Cnr1*+*Vgat* double-labeled cells per mm^2^, and number of *Fos*+*Cnr1*+*Vgat* triple-labeled cells per mm^2^ in OFC. Representative images showing *Fos* (white), *Cnr1* (green), or *Vgat* (red)-expressing cells (20× magnification, scale bar = 25 μm). White arrow denotes *Fos*-positive cell, green arrow denotes *Cnr1*-positive cell, and red arrow denotes *Vgat*-positive cells. Double-labeled cells are denoted by both a white and green arrow. Triple-labeled cells are denoted by a white, green, and red arrow. ***F***, From left to right, Number of *Fos*+ cells per mm^2^, number of *Drd1*+ and *Drd2+* cells per mm^2^, and number of *Fos*+*Drd1* and *Fos+Drd2* double-labeled cells in OFC and Pir. Representative images showing *Fos* (white), or *Drd1* (red), *Drd2* (green; 20× magnification, scale bar = 25 μm). White arrow denotes *Fos*-positive cell, red arrow denotes *Drd1*-positive cell, and green arrow denotes *Drd2*-positive cell. Double-labeled cells are denoted by both a white and green or red arrow (*n* = 6–8 per group); *p ≤ 0.05, different from the No Test group (***E***, ***F***). Data are mean ± SEM. Individual data are shown separately by sex (males = circles, females = triangles) in ***D–F***. OFC, orbitofrontal cortex; Pir, piriform cortex.

**Figure 2.**
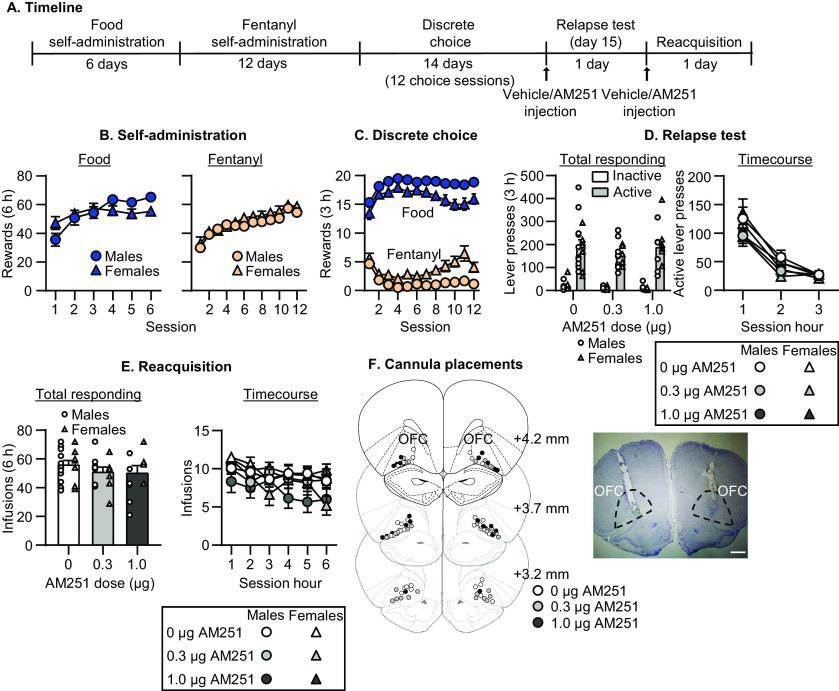
Effect of CB1 receptor blockade in OFC on relapse to fentanyl seeking. ***A***, Timeline of experiment 2. ***B***, Self-administration. Number of reinforced responses (food: 5 pellets/reinforcer; fentanyl 2.5 μg/kg/infusion) during the 6-h sessions. ***C***, Discrete choice (voluntary reduction in self-administration). Number of food-reinforced responses and fentanyl infusions earned during the 3-h choice sessions (20 trials/session). ***D***, Relapse test. Number of active and inactive lever presses during the 3-h test session (left) and 1-h time course (right) after vehicle or AM251 injections (CB1 receptor antagonist). ***E***, Reacquisition test. Number of fentanyl infusions (2.5 μg/kg/infusion) during the 6-h session (left) and 1-h time course (right) after vehicle or AM251 injections in OFC (*n* = 12–20 per dose, between-subjects design). Data are mean ± SEM. Individual data are shown separately by sex (males = circles, females = triangles) in ***D***, ***E***. ***F***, Images showing placement of cannula into OFC at 1.25× magnification (scale bar = 1 mm). Vehicle placements are shown with white circles, 0.3 μg AM251 with gray circles, and 1.0 μg AM251 with black circles.

**Figure 3.**
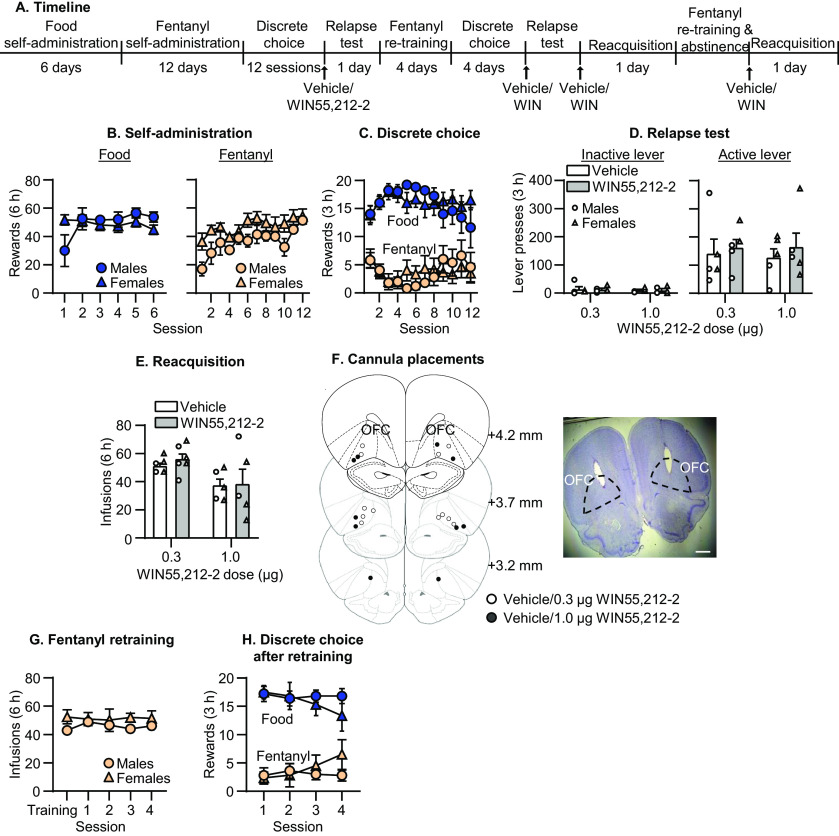
Effect of CB1 receptor agonism in OFC on relapse to fentanyl seeking. ***A***, Timeline of experiment 3. ***B***, Self-administration. Number of reinforced responses (food: 5 pellets/reinforcer; fentanyl 2.5 μg/kg/infusion) during the 6-h sessions. ***C***, Discrete choice (voluntary reduction in self-administration). Number of food-reinforced responses and fentanyl infusions earned during the 3-h choice sessions (20 trials/session). ***D***, Relapse test. Number of inactive (left) and active (right) lever presses during the 3-h test session after vehicle or WIN55,212-2 OFC injections (CB1 receptor agonist). ***E***, Reacquisition test. Number of fentanyl infusions (2.5 μg/kg/infusion) during the 6-h session after vehicle or WIN55,212-2 injections in OFC (*n* = 5 per group in ***D***, *n* = 5–6 per group in ***E***, mixed within/between-subjects design). Data are mean ± SEM. Individual data are shown separately by sex (males = circles, females = triangles) in ***D***, ***E***. ***F***, Images showing placement of cannula into OFC at 1.25× magnification (scale bar = 1 mm). Placements are shown with white (vehicle/0.3 μg WIN55,212-2) or black (vehicle/1 μg WIN55,212-2) circles. ***G***, Mean number of fentanyl infusions during last three sessions of training phase and four sessions of self-administration retraining. ***H***, Number of food and fentanyl rewards during four choice sessions after fentanyl re-training.

**Figure 4.**
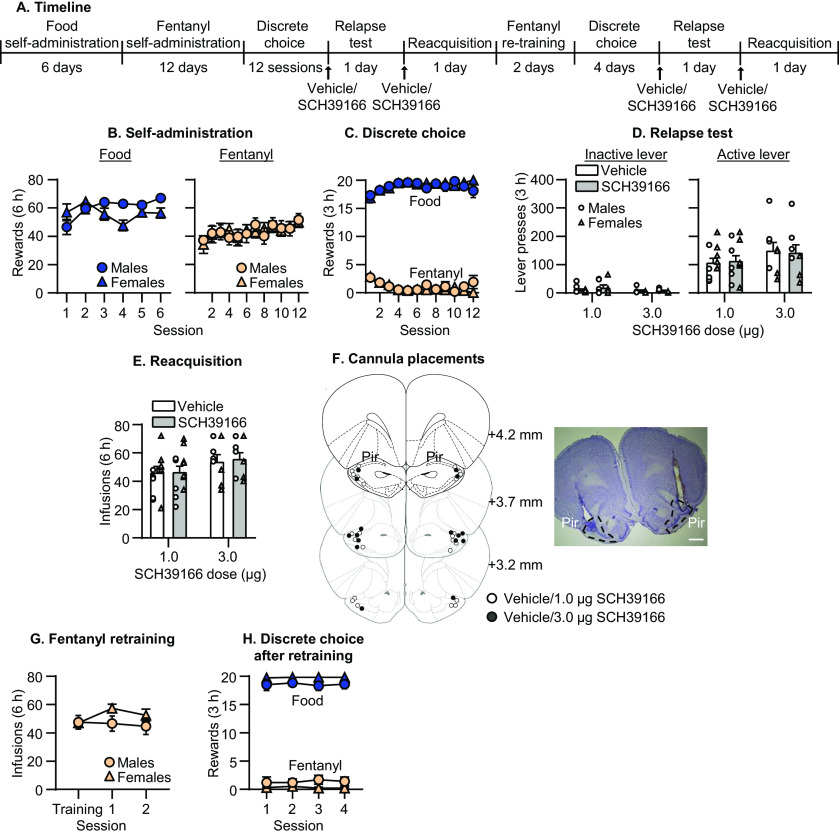
Effect of dopamine D1 receptor blockade in Pir on relapse to fentanyl seeking. ***A***, Timeline of experiment 4. ***B***, Self-administration. Number of reinforced responses (food: 5 pellets/reinforcer; fentanyl 2.5 μg/kg/infusion) during the 6-h sessions. ***C***, Discrete choice (voluntary reduction in self-administration). Number of food-reinforced responses and fentanyl infusions earned during the 3-h choice sessions (20 trials/session). ***D***, Relapse test. Number of inactive (left) and active (right) lever presses during the 3-h test session after vehicle or SCH39166 injections in Pir. ***E***, Reacquisition test. Number of fentanyl infusions (2.5 μg/kg/infusion) during the 6-h session after vehicle or SCH39166 injections in Pir (*n* = 8–11 per group in ***D***, *n* = 8–12 per group in ***E***, mixed within/between-subjects design). Data are mean ± SEM. Individual data are shown separately by sex (males = circles, females = triangles) in ***D***, ***E***. ***F***, Images showing placement of cannula into Pir at 1.25× magnification (scale bar = 1 mm). Placements are shown with white (vehicle/1 μg SCH39166) or black (vehicle/3 μg SCH39166) circles. ***G***, Mean number of fentanyl infusions during last three sessions of training phase and two sessions of self-administration retraining. ***H***, Number of food and fentanyl rewards during four choice sessions after fentanyl retraining.

Based on these considerations, a rat model of relapse after voluntary abstinence was previously developed, achieved by providing rats with a history of drug self-administration mutually exclusive choices between high-carbohydrate palatable food and drug (Caprioli et al., 2015; Venniro et al., 2017a; Fredriksson et al., 2021 but see also Ginsburg and Lamb, 2013 for a similar voluntary abstinence procedure with food vs. alcohol choice). Under this voluntary abstinence procedure, most rats achieve complete fentanyl abstinence during most of the choice sessions (i.e., zero choices of fentanyl infusions). However, in the present study, some rats continue to occasionally self-administer a small number of drug infusions during these sessions (see Figs. 1–4*C*), and we, therefore, refer to the current data as voluntary reduction in self-administration. This discrete choice procedure was used recently to study brain mechanisms of relapse to the potent opioid fentanyl, and the authors focused on orbitofrontal cortex (OFC) because this brain region is critical for relapse to heroin or oxycodone seeking after forced abstinence (Fanous et al., 2012; Altshuler et al., 2021).

In this recent study, the authors first trained male and female rats to self-administer palatable food pellets for 6 d and intravenous fentanyl for 12 d (Reiner et al., 2020). They then assessed relapse to fentanyl seeking after 13–14 voluntary abstinence days, achieved through a discrete choice procedure between fentanyl infusions and palatable food. They found that relapse to fentanyl seeking after food choice-induced voluntary abstinence is associated with increased Fos expression in OFC and that muscimol+baclofen inactivation of OFC decreases relapse to fentanyl seeking (Reiner et al., 2020). They also identified that piriform cortex (Pir) and projections between Pir and OFC are critical for fentanyl relapse (Reiner et al., 2020). These data indicate that both OFC and Pir play a role in fentanyl relapse after food choice-induced abstinence. However, the specific receptor and neurotransmitter mechanisms within OFC and Pir that underlie fentanyl relapse are unknown.

The goal of the current study was two-fold. We first determined whether fentanyl relapse was associated with increased neuronal activity in specific OFC and Pir cell types. We used RNAscope *in situ* hybridization to examine whether neuronal activity (assessed by the neuronal activity marker *Fos*) was increased in OFC and Pir cells expressing cannabinoid 1 (CB1) receptors (assessed by *Cnr1* gene expression), dopamine D1 receptors (*Drd1*), and dopamine D2 receptors (*Drd2*). We chose the CB1 receptor because blockade of these receptors decreases heroin priming-induced and cue-induced reinstatement of heroin seeking (Fattore et al., 2005; Alvarez-Jaimes et al., 2008). We chose the dopamine D1 and D2 receptors because previous studies have shown a role of these receptors in heroin priming-induced, cue-induced, context-induced, and stress-induced reinstatement of heroin seeking and morphine seeking after forced abstinence (Shaham and Stewart, 1996; Shalev et al., 2002; Alvarez-Jaimes et al., 2008; Bossert et al., 2009, 2013; Gao et al., 2013; Lai et al., 2013). However, the causal role of these receptors in OFC and Pir in opioid-relapse-related behaviors is unknown.

We found that fentanyl relapse after food choice-induced reduction in self-administration was associated with increased neuronal activity in OFC CB1 receptor-expressing cells (assessed by co-expression of *Fos* and *Cnr1*) and Pir dopamine D1 receptor-expressing cells (assessed by co-expression of *Fos* and *Drd1*). Importantly, a portion of the OFC CB1 receptor-expressing cells also co-express the GABAergic marker vGAT, indicating that these cells are putative GABAergic interneurons. However, neither injections of the CB1 receptor antagonist AM251 into OFC, the CB1 receptor agonist WIN55,212-2 into OFC, nor injections of the dopamine D1 receptor antagonist SCH39166 into Pir decreased fentanyl relapse after food choice-induced reduction in fentanyl self-administration or reacquisition of fentanyl self-administration.

## Materials and Methods

### Animals

We used 67 male and 67 female Sprague Dawley rats (body weight at the time of intravenous surgery: males, 247–349 g; females, 189–232 g; Charles River). The rats were 8–10 weeks of age at the time of intravenous surgery. We housed the rats two per cage for one to three weeks and then individually after surgery to avoid potential damage to catheter and cannula from social housing. We maintained the rats under a reverse 12/12 h light/dark cycle (lights off at 8 A.M.) with food and water available *ad libitum*. We performed the experiments in accordance with the National Institutes of Health (NIH) *Guide for the Care and Use of Laboratory Animals* (eighth edition). All animal procedures were performed in accordance with NIH regulations and were approved by the institute’s animal care committee. Out of the 134 total rats, we excluded 15 rats because of illness and 4 rats because of loss of catheter patency.

### Drugs

We received fentanyl citrate (fentanyl) from our institutional pharmacy and dissolved it in sterile saline. We chose a unit dose of 2.5 μg/kg/infusion for self-administration training based on a previous study (Reiner et al., 2020). We received the CB1 receptor antagonist AM251 from Sigma (catalog #A6266) and the CB1 receptor agonist WIN55,212-2 from Tocris (catalog #1038) and dissolved them in sterile saline with 8% DMSO, and 5% Tween 80 for intracranial injections. We received the selective dopamine D1 receptor antagonist SCH39166 from Tocris (catalog #2299) and dissolved it in sterile saline.

### Intravenous surgery

We anesthetized the rats with isoflurane gas (5% induction; 2–3% maintenance; Butler Schein) and inserted SILASTIC (VWR) catheters into the jugular vein. We injected the rats with ketoprofen (2.5 mg/kg, s.c.; Butler Schein) 1 h after surgery and the following day to relieve pain and inflammation. We allowed the rats to recover for 5–7 d before food self-administration training. During the recovery and all experimental phases, we flushed the catheters every 24–48 h with gentamicin (4.25 mg/ml; APP Pharmaceuticals) dissolved in sterile saline.

### Intracranial surgery

We performed intracranial surgery in the same session as the intravenous surgery for rats in experiment 2. Using a stereotaxic instrument (Kopf), we implanted guide cannulas (23 gauge; Plastics One) 1 mm above OFC or Pir. We set the nose bar at −3.3 mm and used the following coordinates from bregma: OFC: AP, +3.4 mm; ML, ±3.1 mm (10° angle lateral to midline); DV, −4.9 mm; Pir: AP, +3.4 mm; ML, ±3.9 mm (10° angle lateral to midline); DV, −6.2 mm. We anchored the cannulas to the skull with jeweler’s screws and dental cement.

### Intracranial injections

We injected the CB1 receptor antagonist AM251 or the CB1 receptor agonist WIN55,212-2 into OFC or the dopamine D1 receptor antagonist SCH39166 into Pir 15 min before starting the relapse test sessions. The doses of AM251 (0.3 or 1.0 μg in 0.5 μl/side), WIN55,212-2 (0.3 or 1 μg in 0.5 μl/side), and SCH39166 (1.0 or 3.0 μg in 0.5 μl/side) were based on previous studies (Tan et al., 2011; Caprioli et al., 2017; Venniro et al., 2017b; McReynolds et al., 2018; Doncheck et al., 2020; Rossi et al., 2020; Higginbotham et al., 2021). We injected vehicle or drug at a rate of 0.5 μl/min and left the injectors (which extend 1.0 mm below the tips of the guide cannulas) in place for an additional minute to allow diffusion. We connected the syringe pump (Harvard Apparatus) to 10-μl Hamilton syringes attached to the 30-gauge injectors via polyethylene-50 tubing. We habituated the rats to the injection procedure for 3 d before testing. After testing, we extracted the rats’ brains after isoflurane anesthesia and stored them in 10% formalin. We sectioned the rat brains (50-μm sections) using a Leica cryostat and stained the sections with cresyl violet. Finally, we verified cannula placements under a light microscope. We excluded 24 rats for cannula misplacements.

### RNAscope *in situ* hybridization assay

We performed RNA *in situ* hybridization for *Fos* and *Cnr1*, *Fos*, *Slc32a1*, and *Cnr1*, or *Fos*, *Drd1*, and *Drd2* mRNA. On relapse test day, the rats were either taken from their homecage (No Test, *n* = 6) or were tested for relapse to fentanyl seeking (Test, *n* = 8) and then immediately briefly anesthetized with isoflurane and euthanized. We rapidly extracted and froze the brains for 20 s in −20°C isopentane. We stored the brains at −80°C for further processing. We then collected coronal sections (16 μm) containing the OFC and Pir (+4.2–3.0 mm from bregma) with a cryostat and mounted them directly onto Super Frost Plus slides (Fisher Scientific).

We used RNAscope Multiplex Fluorescent Reagent kit (Advanced Cell Diagnostics) and performed the *in situ* hybridization assay according to the user manual for fresh frozen tissue. We performed three assays, using one section approximately +3.7 to +3.0 mm from bregma for each assay: (1) *Fos* and *Cnr1*; (2) *Fos*, *Slc32a1* (the gene encoding vGAT), and *Cnr1*; and (3) *Fos*, *Drd1*, and *Drd2*. Briefly, on the first day, we fixed the brain sections in 10% neutral buffered formalin (Fisher Scientific) for 20 min at 4°C. We then rinsed the slides three times in PBS and dehydrated them in 50%, 70%, and 100% ethanol. We stored the slides in clean 100% ethanol overnight at −20°C. On the second day, we first dried them at room temperature for 10 min and drew a hydrophobic barrier on slides around brain sections to limit the spreading of the solutions.

We then treated the slides with protease solution (pretreatment 4) at room temperature for 20 min and washed it off. We applied target probes for *Fos* and *Cnr1*, *Fos*, *Cnr1*, and *Slc32a1* (*Vgat*), or *Fos*, *Drd1*, and *Drd2* to the slides and incubated them at 40°C for 2 h in a HybEZ oven. Each target probe contains a mixture of 20 ZZ oligonucleotide probes that are bound to the target RNA: *Fos*-C3 probe (GenBank accession number NM_022197.2; target region, 473–1497; catalog #403591-C3), *Cnr1*-C2 probe (GenBank accession number NM_012784.4; target region, 2–960; catalog #412501-C2), *Slc32a1*-C1 probe (*Vgat*; GenBank accession number NM_031782.1; target region, 288–1666), *Drd1*-C1 probe (GenBank accession number NM_012546.2; target nt region, 104–1053; catalog #317031), and *Drd2*-C2 probe (GenBank accession number NM_012547.1; target nt region, 445–1531; catalog #315641-C2). Next, we incubated the slides with preamplifier and amplifier probes (AMP1, 40°C for 30 min; AMP2, 40°C for 15 min; AMP3, 40°C for 30 min). We then incubated the slides with fluorescently labeled probes by selecting a specific combination of colors associated with each channel: assay 1: green (Alexa 488 nm) for *Cnr1* and far red (Atto 647 nm) for *Fos*, assay 2: green for *Cnr1,* red (Atto 550 nm) for *Slc32a1* (*Vgat*), and far red for *Fos*, or Assay 3: green for *Drd1*, red for *Drd2*, and far red for *Fos*. Finally, we covered the sections with DAPI-containing Vectashield fluorescent mounting medium (H-1400; Vector Laboratories) and cover-slipped them.

### RNAscope *in situ* hybridization quantification

For the RNAscope *in situ* hybridization image acquisition, we used an Olympus VS 120 microscope and captured each image using a 20× objective. We captured one image of Pir or OFC from each hemisphere of one section (+3.7–3.0 mm from bregma) for each assay and used the proximity to the rhinal fissure as a landmark for the 20× images taken of OFC (dorsal and slightly lateral from medial end of rhinal fissure) and Pir (ventral to lateral end of rhinal fissure). We used the Cell Counter tool in ImageJ to manually quantify the total *Fos*-positive cells (at least five white dots surrounding DAPI-positive cells in blue) and the number of *Cnr1*, *Slc32a1*, *Drd1*, and *Drd2-*positive cells (at least five green or red dots surrounding DAPI-positive cells in blue) for OFC or Pir. We also quantified the *Fos*-positive cells co-labeled with *Cnr1*, *Slc32a1*, *Drd1*, or *Drd2*. We performed the image-based quantification in a blind manner with at least two independent counters for each image (mean interrater reliability, *r* = 0.95). The independent counters were blind to the experimental conditions and data reported are from one of the counters.

### Self-administration apparatus

We trained rats to self-administer food and fentanyl in standard self-administration chambers (Med Associates). We equipped each self-administration chamber with two operant panels with three levers located 7–8 cm above the stainless-steel grid floor. We equipped the right panel of the chamber with a discriminative cue (white house light; ENV215M, Med Associates) that signaled the insertion and subsequent availability of the food-paired active (retractable) lever. We equipped the left panel of the chamber with a discriminative cue (red light; ENV-221 M, red lens, Med Associates) that signaled the insertion and subsequent availability of the fentanyl-paired active (retractable) lever. We also equipped the right wall with an inactive (stationary) lever that had no reinforced consequences. We placed a bottle of water and a food hopper with standard laboratory chow on the chamber’s transparent polycarbonate door.

### General procedure

The experiments consisted of three consecutive phases: food self-administration (6 d), fentanyl self-administration (12 d), and choice sessions (12 sessions over 14 d). After the last day of choice, we performed a relapse test. We provide details of the phases and relapse test below.

### Food pellet self-administration training

Before the first self-administration training session, we gave the rats a 1-h magazine-training session, which began with the presentation of the white house light, followed by the noncontingent delivery of one pellet every 3 min. We used 45-mg food pellets (12.7% fat, 66.7% carbohydrate, and 20.6% protein; TestDiet 45-mg pellet, catalog #1811155). We then trained the rats to lever press for food during six 1-h sessions that were separated by 10 min for six consecutive days. The sessions began with the presentation of the white house light, followed 10 s later by the insertion of the food-paired active lever (right panel). The white house light remained on for the duration of the session and served as a discriminative cue for the palatable food. We trained the rats under a fixed-ratio-1 (FR1) 20-s timeout reinforcement schedule, where one lever press resulted in the delivery of five 45-mg palatable food pellets and the presentation of a 20-s discrete tone cue (ENV-223AM, Med Associates), during which additional lever presses were not reinforced but still recorded. At the end of each 1-h session, the white house light was turned off and the active lever was retracted. To match the number of discrete cue presentations to that of fentanyl (see below), we limited the number of food-reinforced deliveries to 12/h.

### Fentanyl self-administration training

We trained rats to self-administer fentanyl during six 1-h daily sessions that were separated by 10 min for 12 d. Fentanyl was infused at a dose of 2.5 μg/kg/infusion over 3.5 s (0.1 ml/infusion). Sessions began with presentation of the red house light for 10 s followed by the insertion of the fentanyl-paired active lever; the red house light remained on for the duration of the session and served as a discriminative cue for fentanyl availability. We trained the rats under an FR1 20-s timeout reinforcement schedule, where one lever press resulted in the delivery of a drug infusion paired with the 20-s discrete white light cue above the fentanyl-paired active lever (ENV-221 M, white lens, Med Associates). At the end of each 1-h session, the red light was turned off and the active lever was retracted. To prevent overdose and decrease self-injurious biting and excessive grooming, we limited the number of infusions to 12/h. In addition, to decrease self-injurious biting, we provided nylabones (Bio-Serv) in the home cage and in the operant chamber beginning with the first day of food self-administration and removed the nylabones from the operant chamber for choice sessions and relapse and reacquisition tests.

### Voluntary reduction in fentanyl self-administration

We conducted 12 discrete choice sessions using the same parameters (dose of fentanyl, number of palatable food pellets per reinforcer delivery, stimuli associated with the two retractable levers) used during the training phases. We divided each 3-h choice session into 20 discrete trials that were separated by 9 min. Each trial began with the presentation of both discriminative cues previously associated with palatable food or fentanyl, followed 10 s later by the insertion of both the palatable food-paired and fentanyl-paired levers. Rats could then select one of the two levers. If the rats responded within 6 min, the reinforcer associated with the selected lever was delivered. Each reinforcer delivery was signaled by the fentanyl-associated or food-associated cue (white cue light or tone, respectively), retraction of both levers, and shutdown of the food and fentanyl discriminative cues. Thus, on a given trial, the rat could earn the drug or food reinforcer, but not both. If a rat failed to respond on either active lever within 6 min, both levers retracted, and their related discriminative cues were shut down with no reinforcer delivery until onset of the next trial.

### Relapse test

The relapse test in the presence of the fentanyl-associated cues consisted of a single 60 min (experiment 1) or 3 h (experiments 2–4) session the day after the last discrete choice session. The session began with the presentation of the red discriminative cue light, followed 10 s later by the insertion of the fentanyl-paired active lever; the red light remained on for the duration of the session. Active lever presses during testing resulted in contingent presentations of the light cue previously paired with drug infusions, but not an infusion of fentanyl. Based on the time course of *Fos* induction (Morgan and Curran, 1991), immediately after the 60-min relapse test of experiment 1, we anesthetized the rats and extracted their brains as described in the next section. For the rats in experiments 2–4, either 2 or 3 d after the relapse test, we tested the rats for reacquisition of fentanyl self-administration using the same parameters as the fentanyl self-administration training.

### Specific experiments

Systemic and intracranial injections of CB1 receptor antagonists or dopamine receptor antagonists decrease heroin priming-induced, context-induced, and cue-induced reinstatement of heroin seeking (Shaham and Stewart, 1996; Shalev et al., 2002; Fattore et al., 2005; Bossert et al., 2007, 2013; Alvarez-Jaimes et al., 2008; See, 2009). In addition, OFC is critical for opioid relapse after forced and voluntary abstinence and Pir is critical for opioid relapse after voluntary abstinence (Fanous et al., 2012; Reiner et al., 2020; Altshuler et al., 2021). We hypothesized that CB1 or dopamine receptors in OFC or Pir play a role in fentanyl relapse. To test this hypothesis, we first determined whether OFC or Pir cells expressing CB1 receptors or dopamine D1 or D2 receptors are activated during the fentanyl relapse test (experiment 1). Next, based on results from experiment 1, we tested the causal role of OFC CB1 receptors (experiments 2 and 3) and Pir dopamine D1 receptors (experiment 4) with intracranial injections of a CB1 receptor antagonist or agonist, or dopamine D1 receptor antagonist, respectively.

### Experiment 1: effect of fentanyl relapse on activity in OFC and Pir cells expressing *Cnr1*, *Drd1*, and *Drd2*

The goal of experiment 1 was to determine whether fentanyl relapse is associated with increased neuronal activity in *Cnr1*, *Drd1*, or *Drd2*-expressing cells in OFC or Pir. In a follow-up assay, we determined whether *Cnr1*-expressing OFC cells co-express *Slc32a1*, a marker of GABAergic interneurons.

We trained male and female rats to self-administer palatable food pellets for 6 d (6 h/d) and fentanyl (2.5 μg/kg/infusion, i.v.) for 12 d (6 h/d). After self-administration, we gave rats 12 choice sessions (20 trials/d). We tested a subset of rats (*n* = 8; 4 males, 4 females) in a 60-min relapse test under extinction conditions. We then euthanized the test rats immediately after the relapse test and the remaining rats (*n* = 6; 3 males, 3 females) as a No Test control group. We extracted the brains and processed the tissue for RNAscope.

### Experiment 2: effect of CB1 receptor blockade in OFC on relapse to fentanyl seeking

The goal of experiment 2 was to determine the causal role of OFC CB1 receptors in fentanyl relapse. We trained rats with cannula targeting OFC as in experiment 1. Before the 3-h relapse test, we injected the rats with the CB1 receptor antagonist AM251 [0 (*n* = 20; 12 males, 8 females), 0.3 (*n* = 14; 8 males, 6 females), or 1 μg (*n* = 12, 6 males, 6 females) per hemisphere] into OFC. Two to 3 d after the relapse test, we tested the effect of OFC CB1 receptor blockade on reacquisition of fentanyl self-administration, using the same doses of AM251. Between the relapse test and reacquisition, we tested the rats in an additional 3-h test under extinction conditions without injections (data not shown). We food restricted five rats during food training for 1–2 d (∼14–16 g of chow pellets overnight) until they acquired palatable food self-administration. During fentanyl self-administration, we accidentally allowed one rat to self-administer 3.45 μg/kg/infusion for the first eight sessions and corrected the dose to 2.5 μg/kg/infusion for the last four sessions. We included this rat in the analysis because there were no differences in the number of fentanyl infusions compared with other rats.

### Experiment 3: effect of CB1 receptor agonism in OFC on relapse to fentanyl seeking

In experiment 2, we found that OFC injections of a CB1 receptor antagonist had no effect on relapse to fentanyl seeking. In experiment 3, we further explored the role of CB1 OFC receptors in relapse by testing the effect of direct stimulation of these receptors by the CB1 receptor agonist WIN55,212-2.

We trained rats with cannula targeting OFC as in experiment 1. Before the 3-h relapse test, we injected the rats with the CB1 receptor agonist WIN55,212-2. We used a mixed within/between-subjects design with WIN55,212-2 Injection as the within-subjects factor and dose as a between-subjects factor [0 and 0.3 μg per hemisphere, within-subjects (*n* = 6; three males, three females); 0 and 1 μg per hemisphere, within-subjects (*n* = 5; 2 males, three females)] into OFC. To perform within-subjects testing on relapse, following the relapse test, we retrained the rats on fentanyl self-administration (four sessions, 6 h/session) and choice (four sessions, 20 trials/session). Data from these sessions did not differ from the last 3 d of fentanyl self-administration in the training phase or from the 12 choice sessions (*p* values > 0.05; Fig. 3*G*). We subsequently completed the mixed within/between-subjects design for the relapse tests, such that rats received both vehicle and either 0.3 or 1 μg WIN55,212-2 (*n* = 6 for vehicle/0.3 μg; *n* = 5 for vehicle/1 μg). We eliminated data from one rat from the relapse test analysis because this rat was a statistical outlier (number of active lever presses was >2 SD above the mean; outlier: 720 lever presses/3 h, mean: 188 lever presses/3 h). Additionally, we confirmed that this rat is an extreme outlier according to the box plot generated with the descriptive statistics feature in SPSS. One day after the last relapse test, we tested the effect of OFC CB1 receptor agonism on reacquisition of fentanyl self-administration, using the same mixed within/between-subjects design and doses of WIN55,212-2. After the first reacquisition test, we retrained the rats on fentanyl self-administration (four sessions, 6 h/session) and choice (four sessions, 20 trials/session), and subsequently re-tested the rats on reacquisition to complete the within-subjects portion of the experiment. Data from these sessions did not differ from the last 3 d of fentanyl self-administration in the training phase or from the 12 choice sessions (*p* values > 0.05; Fig. 3*G*,*H*).

### Experiment 4: effect of dopamine D1 receptor blockade in Pir on relapse to fentanyl seeking

The goal of experiment 4 was to determine the causal role of Pir dopamine D1 receptors in fentanyl relapse. We trained rats with cannula targeting Pir as in experiment 1. Before the 3-h relapse test, we injected the rats with the dopamine D1 receptor antagonist SCH39166 in a mixed within/between-subjects design with SCH39166 injection as the within-subjects factor and dose as the between-subjects factor [0 and 1 μg per hemisphere within-subjects (*n* = 12; 6 males, 6 females); 0 and 3 μg per hemisphere within-subjects (*n* = 8; 4 males, 4 females)] into Pir. Two to 3 d after the relapse test, we tested the effect of Pir dopamine D1 receptor blockade on reacquisition of fentanyl self-administration, using the same dose of SCH39166. To perform within-subjects testing on relapse and reacquisition, following these two tests, we retrained the rats on fentanyl self-administration (two sessions, 6 h/session) and choice (four sessions, 20 trials/session). Data from these sessions did not differ from the last 3 d of fentanyl self-administration in the training phase or from the 12 choice sessions (*p* values > 0.05; Fig. 4*G*,*H*). We subsequently completed the mixed within/between-subjects design for the relapse tests, such that rats received both vehicle and either 1 or 3 μg SCH39166 (*n* = 12 for vehicle/0.3 μg; *n* = 8 for vehicle/1 μg). A subset of these rats (*n* = 3 in the vehicle/1 μg group, *n* = 8 in the vehicle/3 μg group) were tested for reacquisition in the manner described in experiment 3. We eliminated data from one rat from the relapse test analysis because this rat was a statistical outlier (number of active lever presses was >2 SD above the mean; outlier: 761 lever presses/3 h, mean: 126 lever presses/3 h). Additionally, we confirmed that this rat is an extreme outlier according to the box plot generated with the descriptive statistics feature in SPSS.

### Statistical analyses

We analyzed the data with repeated-measures ANOVAs, mixed-factorial ANOVAs, multivariate ANOVAs, and *t* tests using SPSS (version 23, IBM; GLM procedure). We describe the different between-subjects and within-subjects factors for the different statistical analyses in Results. We followed significant main effects and interactions (*p* ≤ 0.05) with *post hoc* PLSD tests. We did not use Sex as a factor in analyses that have a low n per sex per condition (*n* ≤ 5). Additionally, for clarity, we indicate *post hoc* results with asterisks in the figures, but they are not described in Results. For a complete reporting of the statistical analysis, see Table 1.

**Table 1:** Statistical analysis for experiments 1–4 (SPSS GLM repeated-measures module)

Figure number	Factor name	*F* value	*P* value	Partial Eta^2^
Figure 1*B*, self-administration Repeated-measures ANOVA	With sex as a factor Food Sex (male, female), between-subjects Session (1–6), within-subjects Sex × Session interaction Fentanyl Sex (male, female), between-subjects Session (1–12), within-subjects Sex × Session interaction	*F*_(1,12)_ = 1.0 *F*_(5,60)_ = 1.1 *F*_(5,60)_ = 0.1 *F*_(1,12)_ = 0.7 *F*_(11,132)_ = 3.7 *F*_(11,132)_ = 1.4	0.35 0.37 0.99 0.43 <0.001* 0.20	0.07 0.08 0.01 0.05 0.24 0.10
Figure 1*C*, discrete choice Repeated-measures ANOVA	With sex as a factor Preference score Sex (male, female), between-subjects Session (1–12), within-subjects Sex × Session interaction	*F*_(1,12)_ = 4.8 *F*_(11,132)_ = 15.4 *F*_(11,132)_ = 1.0	0.05* <0.001* 0.45	0.28 0.56 0.08
Figure 1*D*, relapse test Total responding Repeated-measures ANOVA	Without sex as a factor Lever (active, inactive), within-subjects	*F*_(1,7)_ = 37.0	<0.001*	0.84
Figure 1*D*, relapse test Time course Repeated-measures ANOVA	Without sex as a factor Session Time (15, 30, 45, 60), within-subjects Lever (active, inactive), within-subjects Session Time × Lever interaction	*F*_(3,21)_ = 11.1 *F*_(1,7)_ = 37.0 *F*_(3,21)_ = 9.6	<0.001* <0.001* <0.001*	0.61 0.84 0.58
Figure 1*F*, Fos neuron counting Repeated-measures ANOVA	OFC Cnr1: without sex as a factor Fos Test Condition (Test, No Test), between-subjects Cnr1 Test Condition (Test, No Test), between-subjects Fos+Cnr1 Test Condition (Test, No Test), between-subjects Pir Cnr1: without sex as a factor Fos Test Condition (Test, No Test), between-subjects Cnr1 Test Condition (Test, No Test), between-subjects Fos+Cnr1 Test Condition (Test, No Test), between-subjects OFC Cnr1 and Vgat: without sex as a factor Cnr1+Vgat Test Condition (Test, No Test), between-subjects Fos+Cnr1+Vgat Test Condition (Test, No Test), between-subjects OFC Drd1 and Drd2: without sex as a factor Fos Test Condition (Test, No Test), between-subjects Drd1 Test Condition (Test, No Test), between-subjects Drd2 Test Condition (Test, No Test), between-subjects Fos+Drd1 Test Condition (Test, No Test), between-subjects Fos+Drd2 Test Condition (Test, No Test), between-subjects Pir Drd1 and Drd2: without sex as a factor Fos Test Condition (Test, No Test), between-subjects Drd1 Test Condition (Test, No Test), between-subjects Drd2 Test Condition (Test, No Test), between-subjects Fos+Drd1 Test Condition (Test, No Test), between-subjects Fos+Drd2 Test Condition (Test, No Test), between-subjects	*F*_(1,12)_ = 10.4 *F*_(1,12)_ = 2.4 *F*_(1,12)_ = 11.7 *F*_(1,12)_ = 5.1 *F*_(1,12)_ = 0.0 *F*_(1,12)_ = 1.6 *F*_(1,12)_ = 0.3 *F*_(1,12)_ = 6.2 *F*_(1,10)_ = 5.4 *F*_(1,10)_ = 2.9 *F*_(1,10)_ = 1.4 *F*_(1,10)_ = 1.6 *F*_(1,10)_ = 2.2 *F*_(1,12)_ = 7.2 *F*_(1,12)_ = 0.0 *F*_(1,12)_ = 1.7 *F*_(1,12)_ = 5.4 *F*_(1,12)_ = 1.7	0.007* 0.15 0.005* 0.04* 0.89 0.23 0.57 0.03* 0.04* 0.12 0.27 0.24 0.17 0.02* 0.99 0.22 0.04* 0.22	0.47 0.17 0.49 0.30 0.00 0.12 0.03 0.34 0.35 0.22 0.12 0.14 0.18 0.37 0.00 0.12 0.31 0.13
Figure 2*B*, self-administration Repeated-measures ANOVA	With sex as a factor Food Sex (male, female), between-subjects Session (1–6), within-subjects Sex × Session interaction Fentanyl Sex (male, female), between-subjects Session (1–12), within-subjects Sex × Session interaction	*F*_(1,44)_ = 0.2 *F*_(5,220)_ = 12.4 *F*_(5,220)_ = 4.6 *F*_(1,44)_ = 0.8 *F*_(11,484)_ = 32.0 *F*_(11,484)_ = 0.7	0.69 <0.001* <0.001* 0.38 <0.001* 0.74	0.00 0.22 0.10 0.02 0.42 0.02
Figure 2*C*, discrete choice Repeated-measures ANOVA	With sex as a factor Preference score Sex (male, female), between-subjects Session (1–12), within-subjects Sex × Session interaction	*F*_(1,44)_ = 12.3 *F*_(11,484)_ = 15.2 *F*_(11,484)_ = 1.7	0.001* <0.001* 0.07	0.22 0.26 0.04
Figure 2*D*, relapse test Total responding Mixed ANOVA	With sex as a factor Sex (male, female), between-subjects AM251 Dose (0, 0.3, 1 μg), between-subjects Lever (active, inactive) within-subjects AM251 Dose × Lever interaction Sex × AM251 Dose interaction Sex × Lever interaction Sex × AM251 Dose × Lever interaction	*F*_(1,40)_ = 0.0 *F*_(2,40)_ = 1.0 *F*_(1,40)_ = 152.7 *F*_(2,40)_ = 0.9 *F*_(2,40)_ = 0.3 *F*_(1,40)_ = 0.0 *F*_(2,40)_ = 1.2	0.94 0.39 <0.001* 0.43 0.74 0.97 0.31	0.0 0.05 0.79 0.04 0.02 0.0 0.06
Figure 2*D*, relapse test Time course Mixed-ANOVA	Without sex as a factor AM251 Dose (0, 0.3, 1 μg), between-subjects Session Hour (1–3) within-subjects Lever (active, inactive), within-subjects AM251 Dose × Session Hour interaction AM251 Dose × Lever interaction Session Hour × Lever interaction AM251 Dose × Session Hour × Lever interaction	*F*_(2,43)_ = 1.1 *F*_(2,86)_ = 144.2 *F*_(1,43)_ = 160.4 *F*_(4,86)_ = 1.8 *F*_(2,43)_ = 1.0 *F*_(2,86)_ = 131.5 *F*_(4,86)_ = 1.4	0.34 <0.001* <0.001* 0.14 0.39 <0.001* 0.24	0.05 0.77 0.79 0.08 0.04 0.75 0.06
Figure 2*E*, reacquisition Mixed-ANOVA	With sex as a factor Sex (male, female), between-subjects AM251 Dose (0, 0.3, 1 μg), between-subjects Session Hour (1–6) within-subjects AM251 Dose × Session Hour interaction Sex × AM251 Dose interaction Sex × Session Hour interaction Sex × AM251 Dose × Session Hour interaction	*F*_(1,40)_ = 1.9 *F*_(2,40)_ = 1.2 *F*_(5,200)_ = 8.7 *F*_(10,200)_ = 1.3 *F*_(2,40)_ = 2.9 *F*_(5,200)_ = 1.9 *F*_(10,200)_ = 1.2	0.18 0.30 <0.001* 0.26 0.07 0.10 0.32	0.04 0.06 0.18 0.06 0.13 0.04 0.06
Figure 3*B*, self-administration Repeated-measures ANOVA	Without sex as a factor Food Session (1–6), within-subjects Fentanyl Session (1–12), within-subjects	*F*_(5,50)_ = 1.5 *F*_(11,110)_ = 5.3	0.22 <0.001*	0.13 0.35
Figure 3*C*, discrete choice Repeated-measures ANOVA	Without sex as a factor Preference score Session (1–12), within-subjects	*F*_(11,110)_ = 2.7	0.004*	0.22
Figure 3*D*, relapse test Total responding Repeated-measures ANOVA	Without sex as a factor (without statistical outlier) WIN55,212-2 Injection (vehicle, WIN55,212-2), within-subjects WIN55,212-2 Dose (0.3, 1 μg), between-subjects Lever (active, inactive) within-subjects WIN55,212-2 Injection × Dose interaction WIN55,212-2 Injection × Lever interaction WIN55,212-2 Dose × Lever interaction WIN55,212-2 Injection × Dose × Lever interaction	*F*_(1,8)_ = 0.4 *F*_(1,8)_ = 0.0 *F*_(1,8)_ = 38.4 *F*_(1,8)_ = 0.0 *F*_(1,8)_ = 0.6 *F*_(1,8)_ = 0.0 *F*_(1,8)_ = 0.0	0.57 0.87 <0.001* 0.86 0.46 0.94 0.85	0.04 0.00 0.83 0.00 0.07 0.00 0.00
Figure 3*D*, relapse test Total responding Repeated-measures ANOVA	Without sex as a factor (with statistical outlier) WIN55,212-2 Injection (vehicle, WIN55,212-2), within-subjects WIN55,212-2 Dose (0.3, 1 μg), between-subjects Lever (active, inactive) within-subjects WIN55,212-2 Injection × Dose interaction WIN55,212-2 Injection × Lever interaction WIN55,212-2 Dose × Lever interaction WIN55,212-2 Injection × Dose × Lever interaction	*F*_(1,9)_ = 0.1 *F*_(1,9)_ = 0.6 *F*_(1,9)_ = 26.5 *F*_(1,9)_ = 0.7 *F*_(1,9)_ = 0.1 *F*_(1,9)_ = 0.5 *F*_(1,9)_ = 0.8	0.75 0.45 <0.001* 0.42 0.73 0.48 0.40	0.01 0.07 0.75 0.07 0.01 0.06 0.08
Figure 3*E*, reacquisition Repeated-measures ANOVA	Without sex as a factor WIN55,212-2 Injection (vehicle, WIN55,212-2), within-subjects WIN55,212-2 Dose (0.3, 1 μg), between-subjects WIN55,212-2 Injection × Dose interaction	*F*_(1,9)_ = 0.6 *F*_(1,9)_ = 4.5 *F*_(1,9)_ = 0.3	0.44 0.06 0.61	0.07 0.33 0.03
Figure 3*G*, re-training Repeated-measures ANOVA	Without sex as a factor Fentanyl Session (1–4), within-subjects	*F*_(3,30)_ = 0.2	0.92	0.02
Figure 3*H*, discrete choice Repeated-measures ANOVA	Without sex as a factor Preference score Session (1–4), within-subjects	*F*_(3,30)_ = 1.5	0.25	0.13
Figure 4*B*, self-administration Repeated-measures ANOVA	With sex as a factor Food Sex (male, female), between-subjects Session (1–6), within-subjects Sex × Session interaction Fentanyl Sex (male, female), between-subjects Session (1–12), within-subjects Sex × Session interaction	*F*_(1,18)_ = 1.3 *F*_(5,90)_ = 3.9 *F*_(5,90)_ = 5.8 *F*_(1,18)_ = 0.0 *F*_(11,198)_ = 2.9 *F*_(11,198)_ = 0.5	0.27 0.003* <0.001* 0.97 0.001* 0.89	0.07 0.18 0.24 0.00 0.14 0.03
Figure 4*C*, discrete choice Repeated-measures ANOVA	With sex as a factor Preference score Sex (male, female), between-subjects Session (1–12), within-subjects Sex × Session interaction	*F*_(1,18)_ = 0.2 *F*_(11,198)_ = 5.9 *F*_(11,198)_ = 1.5	0.66 <0.001* 0.12	0.01 0.25 0.08
Figure 4*D*, relapse test Total responding Repeated-measures ANOVA	Without sex as a factor (without statistical outlier) SCH39166 Injection (vehicle, SCH39166), within-subjects SCH39166 Dose (1, 3 μg), between-subjects Lever (active, inactive) within-subjects SCH39166 Injection × Dose interaction SCH39166 Injection × Lever interaction SCH39166 Dose × Lever interaction SCH39166 Injection × Dose × Lever interaction	*F*_(1,17)_ = 0.0 *F*_(1,17)_ = 0.9 *F*_(1,17)_ = 130.4 *F*_(1,17)_ = 0.2 *F*_(1,17)_ = 0.1 *F*_(1,17)_ = 4.6 *F*_(1,17)_ = 0.0	0.86 0.35 <0.001* 0.65 0.82 0.05* 0.93	0.00 0.05 0.89 0.01 0.00 0.21 0.00
Figure 4*D*, relapse test Total responding Repeated-measures ANOVA	Without sex as a factor (with statistical outlier) SCH39166 Injection (vehicle, SCH39166), within-subjects SCH39166 Dose (1, 3 μg), between-subjects Lever (active, inactive) within-subjects SCH39166 Injection × Dose interaction SCH39166 Injection × Lever interaction SCH39166 Dose × Lever interaction SCH39166 Injection × Dose × Lever interaction	*F*_(1,18)_ = 0.6 *F*_(1,18)_ = 0.0 *F*_(1,18)_ = 44.2 *F*_(1,18)_ = 0.9 *F*_(1,18)_ = 0.3 *F*_(1,18)_ = 0.2 *F*_(1,18)_ = 0.5	0.46 0.99 <0.001* 0.36 0.61 0.68 0.48	0.03 0.00 0.71 0.05 0.02 0.01 0.03
Figure 4*E*, reacquisition Repeated-measures ANOVA	Without sex as a factor SCH39166 Injection (vehicle, SCH39166), within-subjects SCH39166 Dose (1, 3 μg), between-subjects SCH39166 Injection × Dose interaction	*F*_(1,18)_ = 0.2 *F*_(1,18)_ = 1.8 *F*_(1,18)_ = 0.1	0.63 0.20 0.77	0.01 0.09 0.01
Figure 4*G*, re-training repeated-measures ANOVA	With sex as a factor Fentanyl Sex (male, female), between-subjects Session (1–2), within-subjects Sex × Session interaction	*F*_(1,18)_ = 2.2 *F*_(1,18)_ = 1.9 *F*_(1,18)_ = 0.4	0.15 0.19 0.55	0.11 0.09 0.02
Figure 4*H*, discrete choice Repeated-measures ANOVA	With sex as a factor Preference score Sex (male, female), between-subjects Session (1–4), within-subjects Sex × Session interaction	*F*_(1,18)_ = 3.2 *F*_(3,54)_ = 0.1 *F*_(3,54)_ = 0.1	0.09 0.93 0.94	0.15 0.01 0.01

Partial Eta^2^ = proportion of explained variance.

## Results

### Self-administration training and voluntary reduction in self-administration

In both experiments, male and female rats reliably self-administered palatable food and fentanyl (Figs. 1–4*B*) and strongly preferred palatable food over fentanyl during the food versus fentanyl discrete choice sessions (Figs. 1–4*C*). We observed no sex differences in food or fentanyl self-administration in any of the experiments. In experiments 1 and 2, there was a main effect of sex during food choice-induced voluntary reduction in self-administration (Fig. 1*C*, *F*_(1,12)_ = 4.8, *p* = 0.05 and Fig. 2*C*, *F*_(1,44)_ = 12.3, *p* = 0.001), with female rats showing slightly decreased food preference compared with male rats. There was no effect of sex during the choice sessions in experiment 4 (Fig. 4*C*, *F*_(1,18)_ = 0.2, *p* = 0.66). For experiments 1, 2, and 4, the mean ± SEM number of fentanyl infusions during the 12 choice sessions (20 trials/d) was 0.94 ± 0.55, 1.45 ± 0.44, and 1.11 ± 0.61 for males, and 1.38 ± 0.72, 3.71 ± 0.88, and 0.88 ± 0.43 for females. Because of low n per sex (*n* ≤ 5), we do not use Sex as a factor in the analyses of the relapse and RNAscope data in experiment 1, the behavioral data in experiment 3, and the relapse and reacquisition data of experiment 4. We also show data for male and female rats in line graphs and individual data from male and female rats in bar graphs.

### Experiment 1: effect of fentanyl relapse on activity in OFC and Pir cells expressing *Cnr1*, *Drd1*, and *Drd2*

The goal of experiment 1 was to determine whether relapse to fentanyl seeking is associated with increased neuronal activity in *Cnr1*, *Drd1*, or *Drd2-*expressing OFC or Pir cells. The timeline of experiment 1 is provided in Figure 1*A*.

### Relapse test (day 15)

The number of lever presses on the active lever was greater than the number of lever presses on the inactive lever during relapse to fentanyl seeking (Fig. 1*D*, left). The repeated-measures ANOVA for total number of lever presses showed a significant effect of Lever (*F*_(1,6)_ = 39.9, *p* < 0.001). For the time course of lever presses (Fig. 1*D*, right), the repeated-measures ANOVA included the within-subjects factors of Session Time (15, 30, 45, 60 min) and Lever. The analysis showed a significant interaction between the two factors (*F*_(3,21)_ = 9.6, *p* < 0.001).

### RNAscope quantification for *Fos*+*Cnr1* in OFC and Pir

We quantified the number of OFC and Pir *Fos*-positive, *Cnr1*-positive, and *Fos*+*Cnr1* double-labeled cells after the day 15 relapse test (Fig. 1*E*). We analyzed each brain region with separate repeated-measures ANOVAs that included the between-subjects factor of Test Condition (No Test, Test). For CB1 receptor expression in OFC, the analysis showed a significant effect of Test Condition for *Fos* (*F*_(1,13)_ = 10.4, *p* = 0.007) and *Fos*+*Cnr1* (*F*_(1,12)_ = 11.7, *p* = 0.005) but not *Cnr1* (*F*_(1,12)_ = 2.4, *p* = 0.15). To determine whether *Cnr1*-expressing OFC cells co-express *Slc32a1* (the gene that encodes vGAT) and are putative GABAergic interneurons, we ran a second assay for *Cnr1*, *Slc32a1*, and *Fos*. We found that ∼17–20% of OFC *Cnr1*-expressing cells co-express *Slc32a1* (No Test: 19 ± 4 *Cnr1*+*Slc32a1* cells out of a total of 91 ± 5 *Cnr1* cells; Test: 22 ± 3 *Cnr1*+*Slc32a1* cells out of a total of 127 ± 14 *Cnr1* cells). For CB1 receptor expression on GABAergic OFC neurons, the analysis showed no significant effect of Test Condition for *Cnr1*+*Slc32a1* (*F*_(1,12)_ = 0.3, *p* = 0.57) but a significant effect of Test Condition for *Fos*+*Cnr1*+*Slc32a1* (*F*_(1,12)_ = 6.2, *p* = 0.03). For CB1 receptor expression in Pir, the analysis showed a significant effect of Test Condition for *Fos* (*F*_(1,12)_ = 5.1, *p* = 0.04) but not *Cnr1* (*F*_(1,12)_ = 0.0, *p* = 0.89) or *Fos*+*Cnr1* (*F*_(1,12)_ = 1.6, *p* = 0.23).

### RNAscope quantification for *Fos*+*Drd1* or *Drd2* in OFC and Pir

We quantified the number of OFC and Pir *Fos*-positive, *Drd1*-positive, *Drd2*-positive, and *Fos*+*Drd1* and *Fos+Drd2* co-labeled cells after the day 15 relapse test (Fig. 1*F*). For dopamine receptor expression in OFC, the analysis showed a significant effect of Test Condition for *Fos* (*F*_(1,10)_ = 5.4, *p* = 0.04) but not *Drd1* (*F*_(1,10)_ = 2.9, *p* = 0.12), *Drd2* (*F*_(1,10)_ = 1.4, *p* = 0.27), *Fos*+*Drd1* (*F*_(1,10)_ = 1.6, *p* = 0.24), or *Fos*+*Drd2* (*F*_(1,10)_ = 2.2, *p* = 0.17). For dopamine receptor expression in Pir, the analysis showed a significant effect of Test Condition for *Fos* (*F*_(1,12)_ = 7.2, *p* = 0.02) and *Fos*+*Drd1* (*F*_(1,12)_ = 5.4, *p* = 0.04), but not *Drd1* (*F*_(1,12)_ = 0.0, *p* = 0.99), *Drd2* (*F*_(1,12)_ = 1.7, *p* = 0.22), or *Fos+Drd2* (*F*_(1,12)_ = 1.7, *p* = 0.22).

Taken together, these data show that relapse to fentanyl seeking was associated with increased *Fos* expression in *Cnr1*-expressing OFC cells, a portion of which co-express *Slc32a1* and are putative GABAergic interneurons, and in *Drd1*-expressing Pir cells.

### Experiment 2: effect of CB1 receptor blockade in OFC on relapse to fentanyl seeking

In experiment 1, we found that relapse to fentanyl seeking was associated with activation of *Cnr1*-expressing cells in OFC. The goal of experiment 2 was to determine whether CB1 receptors in OFC play a causal role in relapse using OFC injections of the CB1 receptor antagonist AM251. The timeline of experiment 2 is provided in Figure 2*A*.

#### Relapse test

AM251 injections into OFC had no effect on relapse to fentanyl seeking (Fig. 2*D*, left). The mixed ANOVA for total number of lever presses included the between-subjects factors of AM251 Dose (0, 0.3, 1 μg AM251) and Sex and the within-subjects factor of Lever. The analysis showed a significant effect of Lever (*F*_(1,40)_ = 152.7, *p* < 0.001), but no significant effect of AM251 Dose (*F*_(2,40)_ = 1.0, *p* = 0.39) or Sex (*F*_(1,40)_ = 0.0, *p* = 0.94), and no interactions between any of the factors (*p* values > 0.05). For the time course of lever presses (Fig. 2*D*, right), the mixed ANOVA included the between-subjects factor of AM251 Dose and the within-subjects factors of Session Hour (1–3) and Lever. The analysis showed significant effects of Session Hour (*F*_(2,86)_ = 144.2, *p* < 0.001), Lever (*F*_(1,43)_ = 160.4, *p* < 0.001), and an interaction between the two factors (*F*_(2,86)_ = 131.5, *p* < 0.001). There was no significant effect of AM251 Dose (*F*_(2,43)_ = 1.1, *p* = 0.34) or an interaction with any of the other factors (*p* values > 0.05).

#### Reacquisition test

AM251 injections into OFC had no effect on reacquisition of fentanyl self-administration (Fig. 2*E*). The mixed ANOVA included the between-subjects factors of AM251 Dose and Sex and the within-subjects factor of Session Hour (1–6). The analysis showed a significant effect of Session Hour (*F*_(5,200)_ = 5.7, *p* < 0.001) but not AM251 Dose (*F*_(2,40)_ = 1.2, *p* = 0.30), Sex (*F*_(1,40)_ = 1.9, *p* = 0.18), or an interaction between the factors (*p* values > 0.05).

Taken together, these data show that OFC CB1 receptor blockade had no effect on relapse to fentanyl seeking or on reacquisition to fentanyl self-administration.

### Experiment 3: effect of CB1 receptor agonism in OFC on relapse to fentanyl seeking

In experiment 2, we found no effect of CB1 receptor blockade in OFC on fentanyl relapse. The goal of experiment 3 was to determine the effect of activation of CB1 receptors in OFC on relapse with OFC injections of the CB1 receptor agonist WIN55,212-2. The timeline of experiment 3 is provided in Figure 3*A*.

#### Relapse test

WIN55,212-2 injections into OFC had no effect on relapse to fentanyl seeking (Fig. 3*D*). The mixed ANOVA for total number of lever presses included the between-subjects factor of WIN55,212-2 Dose (0.3, 1.0 μg) and the within-subjects factors of WIN55,212 Injection (vehicle, WIN55,212-2) and Lever. The analysis showed a significant effect of Lever (*F*_(1,8)_ = 38.4, *p* < 0.001), but no significant effect of WIN55,212-2 Dose (*F*_(1,8)_ = 0.0, *p* = 0.87) or Injection (*F*_(1,8)_ = 0.4, *p* = 0.57), and no interactions between any of the factors (*p* values > 0.05). Inclusion of a statistical outlier did not change the outcome of the analyses (see Table 1).

#### Reacquisition test

WIN55,212-2 injections into OFC had no effect on reacquisition of fentanyl self-administration (Fig. 3*E*). The mixed ANOVA included the between-subjects factor of WIN55,212-2 Dose and the within-subjects factor of WIN55,212 Injection. The analysis showed no significant effects of WIN55,212-2 Dose (*F*_(1,9)_ = 4.5, *p* = 0.06) or Injection (*F*_(1,9)_ = 0.6, *p* = 0.44), and no interaction between the factors (*p* values > 0.05).

Taken together, these data show that OFC CB1 receptor agonism had no effect on relapse to fentanyl seeking or on reacquisition to fentanyl self-administration.

### Experiment 4: effect of dopamine D1 receptor blockade in Pir on relapse to fentanyl seeking

In experiment 1, we found that relapse to fentanyl seeking was associated with activation of *Drd1*-expressing cells in Pir. The goal of experiment 4 was to determine whether dopamine D1 receptors in Pir play a causal role in relapse using Pir injections of the dopamine D1 receptor antagonist SCH39166. The timeline of experiment 4 is provided in Figure 4*A*.

#### Relapse test

SCH39166 injections into Pir had no effect on relapse to fentanyl seeking (Fig. 4*D*). The mixed ANOVA for total number of lever presses included the between-subjects factor of SCH39166 Dose (1.0, 3.0 μg) and the within-subjects factors of SCH39166 Injection (vehicle, SCH39166) and Lever. The analysis showed a significant effect of Lever (*F*_(1,17)_ = 130.4, *p* < 0.001), but no significant effect of SCH39166 Dose (*F*_(1,17)_ = 0.9, *p* = 0.35) or Injection (*F*_(1,17)_ = 0.0, *p* = 0.86). The analysis showed a significant Dose × Lever interaction (*F*_(1,17)_ = 4.6, *p* = 0.05) but no interactions between any of the other factors (*p* values > 0.05). Inclusion of a statistical outlier did not change the outcome of the analyses, except that the Dose × Lever interaction was no longer statistically significant (see Table 1).

#### Reacquisition test

SCH39166 injections into Pir had no effect on reacquisition of fentanyl self-administration (Fig. 4*E*). The mixed ANOVA included the between-subjects factor of SCH39166 Dose and the within-subjects factor of SCH39166 Injection. The analysis showed no significant effects of SCH39166 Dose (*F*_(1,18)_ = 1.8, *p* = 0.20) or Injection (*F*_(1,18)_ = 0.2, *p* = 0.63), and no interaction between the factors (*p* values > 0.05).

Taken together, these data show that Pir dopamine D1 receptor blockade had no effect on relapse to fentanyl seeking or on reacquisition to fentanyl self-administration.

## Discussion

A previous study showed that OFC and Pir play critical roles in fentanyl relapse after food choice-induced voluntary abstinence (Reiner et al., 2020). Here, we determined the role of cannabinoid receptors in OFC and dopamine receptors in Pir in fentanyl relapse. Using RNAscope *in situ* hybridization, we observed that fentanyl relapse was associated with activation of CB1 receptor-expressing cells in OFC and dopamine D1 receptor-expressing cells in Pir. However, injections of the CB1 receptor antagonist AM251 or CB1 receptor agonist WIN55,212-2 into OFC or the dopamine D1 receptor antagonist SCH39166 into Pir had no effect on fentanyl relapse or reacquisition of fentanyl self-administration. Together, these data suggest that, despite anatomical evidence, pharmacological manipulations do not support causal roles of OFC CB1 receptors or Pir dopamine D1 receptor in fentanyl relapse.

### Anatomical evidence for OFC *Cnr1* and Pir *Drd1* in fentanyl relapse: RNAscope data

We observed that fentanyl relapse after food choice-induced reduction in fentanyl self-administration was associated with increased *Fos* mRNA expression in OFC and Pir using RNAscope *in situ* hybridization. These results are in agreement with a previous study showing that fentanyl relapse is associated with increased Fos protein expression in OFC and Pir (Reiner et al., 2020). The Pearson’s correlation of *Fos* expression in OFC or Pir with fentanyl relapse-responding shows inconsistent results across multiple RNAscope assays (OFC: *r* = 0.17, −0.65, 0.08; Pir: −0.36, −0.19). However, these data should be interpreted with caution because in each assay, *Fos* expression was only examined at a single 20× field of view at a single anterior-posterior plane and thus does not represent a comprehensive analysis of Fos expression throughout OFC and Pir.

We report similar expression of *Cnr1* in OFC and Pir, higher expression of *Drd1* in Pir than OFC, and very low *Drd2* expression in Pir. Within OFC, we report that ∼15% of OFC *Fos+* cells co-express *Cnr1*. Because CB1 receptors are expressed presynaptically, we then examined whether *Cnr1*-expressing cells co-express *Slc32a1* (the gene that encodes vGAT) and are putative GABAergic interneurons. Approximately 20% of *Cnr1*-expressing cells co-express *Slc32a1*, and ∼4% of *Fos*-expressing OFC cells co-express both *Cnr1* and *Slc32a1*. Within Pir, 15% of Pir *Fos+* cells co-express *Drd1*. Together, these data provide anatomical evidence for a role of OFC CB1 receptors and Pir dopamine D1 receptor in fentanyl relapse.

### Lack of effect of CB1 receptor blockade or agonism in OFC on fentanyl relapse

Based on the RNAscope data showing that fentanyl relapse is associated with activation of OFC CB1 receptor-expressing cells, we hypothesized that blockade of OFC CB1 receptors would decrease fentanyl relapse. Our hypothesis was based on previous studies showing that systemic injections of a CB1 receptor antagonist decreases heroin priming-induced and cue-induced reinstatement of heroin seeking and that CB1 receptor blockade in prefrontal cortex and nucleus accumbens decreases cue-induced reinstatement of heroin seeking (Fattore et al., 2005; Alvarez-Jaimes et al., 2008). However, we did not observe an effect of OFC injections of the CB1 receptor antagonist AM251 on relapse to fentanyl seeking. CB1 receptors inhibit release and blockade of these receptors may have a downstream impact on endocannabinoid tone, which could have confounded our results. Based on this consideration, we also determined the effect of direct activation of OFC CB1 receptors on relapse, using the CB1 receptor agonist WIN55,212-2. In this experiment, we also did not observe an effect of OFC CB1 receptor agonism on fentanyl relapse. Together, these results indicate OFC CB1 receptors do not play a role in relapse to fentanyl seeking after voluntary reduction in self-administration.

### Lack of effect of dopamine D1 receptor blockade in Pir on fentanyl relapse

Based on the RNAscope data showing that fentanyl relapse is associated with activation of Pir dopamine D1 receptor-expressing cells, we hypothesized that blockade of Pir dopamine D1 receptors would decrease fentanyl relapse. To the best of our knowledge, there are no previous studies on the role of dopamine transmission in Pir in relation to drug taking-related or seeking-related behaviors. Previous studies have shown a role of dopamine D1 receptors in heroin priming-induced, cue-induced, context-induced, and stress-induced reinstatement of heroin seeking and morphine seeking after forced abstinence (Shaham and Stewart, 1996; Shalev et al., 2002; Bossert et al., 2009, 2013; Gao et al., 2013; Lai et al., 2013). However, we did not observe an effect of Pir injections of the dopamine D1 receptor antagonist SCH39166 on relapse to fentanyl seeking after food choice-induced voluntary reduction in self-administration.

### Potential reasons for lack of effect of the pharmacological manipulations on relapse to fentanyl seeking

We used an approach similar to previous studies using RNAscope *in situ* hybridization and intracranial pharmacology to identify causal roles of neurotransmitter receptors in relapse to drug seeking (Li et al., 2015; Caprioli et al., 2017; Venniro et al., 2017b; Rossi et al., 2020). We describe three potential reasons why we did not observe an effect of our pharmacological manipulations on relapse to fentanyl seeking despite anatomic evidence with RNAscope *in situ* hybridization.

The first reason could be that the doses of AM251 and SCH39166 used in our studies were too low to observe a behavioral effect. Injections of the lower dose of AM251 used in our study (0.3 μg/hemisphere) into the prelimbic cortex decrease the potentiation of cocaine priming-induced reinstatement by intermittent footshock or corticosterone (McReynolds et al., 2018; Doncheck et al., 2020). Injections of WIN55,212-2 within the dose range used in our study (0.3–1.0 μg/hemisphere) into basolateral amygdala increase acquisition of fear conditioning (Tan et al., 2011). Additionally, injections of the lower dose of SCH39166 we used in our study (1 μg/hemisphere) into central amygdala, dorsomedial striatum, or nucleus accumbens core decrease relapse to methamphetamine seeking after food choice-induced voluntary abstinence (Caprioli et al., 2017; Venniro et al., 2017b; Rossi et al., 2020). Together, we used similar or higher doses of pharmacological agents as previous studies that reported effects on different forms of learned behaviors, including drug relapse/reinstatement. Therefore, it seems unlikely that the doses of AM251, WIN55,212-2, or SCH39166 used here were too low to have behavioral effects. However, we cannot rule out this possibility because of potential differences in dose efficacy when injected into different brain regions.

The second reason is that pharmacological manipulations only block activity at the level of the respective receptor, which may lead to changes in downstream intracellular signaling but do not selectivity and directly change the activity of Fos-positive cells during the relapse test. AM251 blocked CB1 receptors in OFC but did not directly inhibit the activity of OFC CB1 receptor-expressing cells that were activated during the relapse test. Importantly, this approach assumes that at least a portion of CB1 receptors, which are presynaptic, are expressed in OFC, presumably on GABAergic interneurons. Therefore, an important caveat of our study is that ∼20% of *Cnr1*-expressing OFC cells co-express *Slc32a1* (the gene that encodes vGAT) and are thus putative GABAergic neurons that would be affected by OFC injections of AM251 or WIN55,212-2. The remaining ∼80% of OFC *Cnr1*-expressing cells are likely to be glutamatergic projection neurons with CB1 receptor protein expression at the axon terminals in OFC output regions and would not be directly impacted by pharmacological manipulations in OFC.

The third reason is that the pharmacological manipulations were not effective because they only modulated the activity of a small proportion of the relapse-associated activated (Fos-positive) cells. In this regard, we found that only ∼15% of *Fos*-positive cells in OFC and Pir co-express *Cnr1* and *Drd1*, respectively (Fig. 1*E*,*F*). In contrast, in previous studies using RNAscope in which intracranial dopamine receptor antagonists decreased relapse to drug seeking, ∼50% of the *Fos*-positive cells co-expressed *Drd1* or *Drd2* in amygdala and striatal regions (Li et al., 2015; Caprioli et al., 2017; Venniro et al., 2017b; Rossi et al., 2020). We speculate that for relapse-related behavioral effects to be observed with pharmacological blockade there needs to be 50% or more *Fos*-positive relapse-associated activated cells that express the receptor targeted by the pharmacological manipulation.

### Methodological considerations

There are several methodological considerations to consider in our study. First, we did not include a positive behavioral or anatomic control to ensure that intracranial administration of the compounds used in our study was successful. However, the current methods are the same as in our previous studies in which we observed behavioral effects of intracranial administration of different pharmacological agents (Venniro et al., 2017b; Reiner et al., 2020). We frequently checked the patency of the needles and tubing in our set up throughout the injection procedure. Thus, while we are confident that we successfully administered the intracranial injections, we cannot rule out the possibility of an experimental issue during the drug preparations and infusions.

The second limitation is a low n per group in experiment 3. Despite the lack of effect of WIN55,212-2 OFC injections on fentanyl relapse, it is possible that the low n in this experiment and individual variability in the data may confound interpretation of the data. Therefore, the results of experiment 3 should be interpreted with caution.

Finally, some rats continued to occasionally self-administer a low level of fentanyl during the discrete food versus fentanyl choice sessions, and thus did not achieve complete abstinence. We therefore refer to the current data during the choice sessions as voluntary reduction in self-administration and acknowledge that low levels of drug infusions can have an impact on opioid receptor regulation and related neuroadaptations.

In conclusion, fentanyl relapse after food choice-induced voluntary reduction in self-administration was associated with activation of CB1 receptor-expressing OFC cells and dopamine D1 receptor-expressing Pir cells, but pharmacological manipulations do not support causal roles of OFC CB1 receptors or Pir dopamine D1 receptors in fentanyl relapse. Our findings highlight the importance of following up correlational anatomic studies with experiments to determine causal mechanisms of relapse to drug-seeking.

## Synthesis

Reviewing Editor: Christie Fowler, University of California, Irvine Decisions are customarily a result of the Reviewing Editor and the peer reviewers coming together and discussing their recommendations until a consensus is reached. When revisions are invited, a fact-based synthesis statement explaining their decision and outlining what is needed to prepare a revision will be listed below. The following reviewer(s) agreed to reveal their identity: Olivier George.

In the reviews and further consultation, both reviewers were positive regarding the potential for the data to provide important information for the field. Building on prior findings that the OFC and piriform cortex are involved in fentanyl relapse, the paper examines potential mechanisms and finds a surprising disconnection between cellular activation and functional mechanism. The topic was described as significant, novel and timely, the design, approach and analyses are rigorous and the conclusions are clear. The model (choice-induced voluntary abstinence) is appropriate. However, several concerns remain, which need to be addressed:

1. The use of the term voluntary abstinence in the text and the title misleading as some of the animals continue to self-administer fentanyl, albeit at low levels, during the so called “voluntary abstinence”. This term should not be used to describe the data. On average these animals self-administer 5 rewards at the end of the protocol, this is significant level of self-administration that will have an important impact on opioid receptor regulation and related neuroadaptations compared to zero infusion (abstinence). This is particularly clear in Fig 2C and 3C. The animals who self-administer at low levels should be removed or analyzed separately, or the authors should change the interpretation of their paradigm by using the term “voluntary reduction in self-administration”.

2. There was some concern of the low n per group in Experiment 3, especially given the variance in this task. Both reviewers felt that this concern regarding the experiment potentially being underpowered could be addressed in the discussion in an ‘Limitations’ paragraph.

3. There is no positive control to ensure that the intracerebral administration of the compounds was successful. There is no measure of Fos activation/inactivation (e.g. fos plumes) and no behavioral changes that can serve as positive control. While the methods seem appropriate, without such control it is impossible to rule out a possible experimental issue during the infusion/drug preparation. Both reviewers felt that the concern could be addressed in the discussion in an ‘Limitations’ paragraph.

4. Highly significant sex differences are stated in the text (p12 L317) that point to figures that do not show sex groups (Fig 1C, 2C), This is odd, either show the male/female in the amin figure or in supplemental figures. Also, sex is not depicted in the plots. It would be useful for the reader to have 2 types of individual markers (different color/shape) for male and female throughout the results.
